# The Clinical Application of One-Stop Examination with 640-Slice Volume CT for Nutcracker Syndrome

**DOI:** 10.1371/journal.pone.0074365

**Published:** 2013-09-16

**Authors:** Jianguo Zhong, Jianhua Yuan, Vincent Chong, Zhen Wang, Jian Xu, Zhongxiang Ding

**Affiliations:** 1 Department of Radiology, Zhejiang Provincial People’s Hospital, Hangzhou, Zhejiang, P. R. China; 2 Department of Diagnostic Imaging, National University Health System, Yong Loo Lin School of Medicine, National University of Singapore, Singapore; University of Washington School of Medicine, United States of America

## Abstract

**Background:**

To investigate the relationship between the level of left renal vein (LRV) compression and changes in the perfusion of the left kidney in patients with nutcracker syndrome (NCS) by one-stop whole-organ perfusion imaging of bilateral kidneys using 640-slice volume CT.

**Methods:**

Twelve patients, clinically diagnosed with NCS, were subjected to one-stop examination of kidneys. Angiography and whole-organ perfusion imaging of bilateral kidneys were conducted, and the compression segment of LRV was demonstrated and measured. Information including the results of whole-organ perfusion images of both kidneys in 12 patients was collected. Results of epigastrium dynamic volume scanning by 640-slice volume CT were collected for 12 patients as control group. Left and right renal cortexes were chosen as regions of interest (ROI), and their perfusion values were measured.

**Results:**

The perfusion values of the left and right renal cortexes in the control group were 323.8 ml·min^−1^·100 ml^−1^ and 322.9 ml·min^−1^·100 ml^−1^, respectively. The difference was not statistically significant (*t = *1.388, *P = *0.193). For NCS patients, the perfusion values of the left and right renal cortexes were 350.8 ml·min^−1^·100 ml^−1^ and 391.1 ml·min^−1^·100 ml^−1^, respectively. Significantly decreased value was observed in left renal cortex compared to that of the right renal cortex, with the mean decrease of 40.3 ml·min^−1^·100 ml^−1^, and the difference was statistically significant (*t* = −4.204, *P = *0.001).

**Conclusion:**

As a non-invasive functional imaging technique, whole-organ perfusion imaging of kidneys can be used to evaluate the organ and tissue perfusion status and to accurately reflect the hemodynamic changes of the left renal cortex in the patients with NCS. Whole organ perfusion imaging may also provide the basis for quantitative diagnosis and clinical interventions of NCS.

## Introduction

Nutcracker syndrome (NCS), also known as renal vein entrapment syndrome, refers to compression of the left renal vein (LRV) that is most commonly seen between the abdominal aorta (AA) and the superior mesenteric artery (SMA) resulting in impaired blood outflow. The radiological evaluation plays a critical role in the diagnosis of NCS, and has been considered as an essential method in clinical intervention in NCS. However, most of the studies have focused on the diagnostic value of different imaging methods in NCS [1],[2],[3],[4],[5],[6],[7]. There is a lack of studies investigating the hemodynamic changes following LRV compression, or evaluating the perfusion level and renal functions. CT perfusion imaging is one of the most important techniques of functional CT imaging, that providing a non-invasive platform for the investigation of renal blood flow abnormalities [8], [9]. The 640-slice volume CT system extends the z-axis coverage to 16 cm and can correlate the physiological functions of organs with their morphological changes making the examination an easy “one-stop” study. In the present study, one-stop examination with 640-slice volume CT was performed and various restructuring methods were used to exhibit the relationship among LRV, SMA and AA. Data of the whole-organ perfusion imaging of both kidneys were collected to evaluate the relationship between the level of LRV compression and changes in perfusion of left kidney in patients with NCS.

## Methods

### General Information

Twelve patients were diagnosed with NCS based on typically clinical manifestation and radiological findings. All 12 patients were subjected to one-stop examination with 640-slice volume CT. There were 9 males and 3 females (age range, 14–63 years, mean age, 27.8 years). The length of time between the initial clinical diagnosis to the present study ranged between half a month to eleven years. Ten patients had gross hematuria which was aggravated by motion; one patient was found to have albuminuria; two patients had microscopic hematuria and 4 patients presented with left-sided waist pain. No symptoms of hypertension, urinary frequency or dysuria were found. The serum creatinine (Scr) and blood urea nitrogen (BUN) were normal. All 12 patients underwent color doppler ultrasonography and the results indicated LRV compression and stricture at the angle of SMA. The inner diameters of divergent part and compression part of LRV were 8.74±1.50 and 2.74±0.93 mm respectively, and the LRV diameter ratio was 3.44±1.08. Peak velocity (PV) was measured in the transverse plane at two points in the LRV, one at the lateral portion of the LRV near the hilum; and the other where the LRV courses between the AA and SMA. The mean velocity of blood flow was 22.9±6.5 and 126.1±33.2 cm/s respectively, and the ratio was 5.84±2.13. Urethrocystoscopy was performed in all 12 patients and seven patients showed hematuria from the left ureter orifice. The delayed venous phase of the left renal arteriogram showed LRV stricture in the angle between SMA and AA, the inner diameters of divergent part and compression part of LRV was 11.10±1.81 and 3.58±1.38 mm respectively, and the LRV diameter ratio was 3.53±1.35. Among these 12 patients, peri-renal and peri-ureteral venous collaterals were shown (four of these patients showed LRV stricture of more than 75%, one patient showed LRV stricture of 50%–75%). Engorged gonadal vein varices were shown in 2 cases with LRV stricture of more than 75%, 1 case with 50%–75%, and 1 case with mild stenosis. Five of these 12 patients underwent laparoscopic stent implantation of LRV. The laparoscopic observation further confirmed the nutcracker syndrome. All 12 patients were diagnosed as NCS and none of them had urologic diseases (such as calculus, tumor, deformity or infection), vascular malformation or vessel-involving systemic diseases.

This study was approved by ethics committee of Zhejiang Provincial People’s Hospital. All the patients or parents of children participants signed informed consent. Data of epigastrium dynamic volume scanning by 640-slice volume CT of the patients in our hospital were retrospectively analyzed. The exclusion criteria included: diseases, which involved hemodynamic changes of renal arteries or veins (such as heart failure); congenital kidney malformations such as horseshoe kidney; a history of serious allergic reaction and intolerance to iodinated contrast agents. No serious medical history was noted in these patients. Finally there were 31 patients that were eligible for inclusion in the study and 12 patients who were age- and sex- matched to the NCS group were chosen as control group (9 males and 3 females) aged 17–59 years old with the mean age of 30.6 years.

### Parameters and Process of CT Scanning

Aquilion One 640-slice volume CT (Toshiba, Japan) was used for the scanning. All the patients fasted for 6-12-hour and avoided intense activities for 30 min before the examination.

### Scanning Process

Low dose acquisition mode of volume CT scanning was used. The parameters of the volume CT scanning included: 100 kV, 75 or 100 mA with the use of 320 detectors, each with a 0.5 mm thickness. The examining table remained immobile during the scanning. The settings included: 0.5 s for the turb to rotate one cycle, field of view of 350×350 mm, matrices of 512×512 and the z-direction coverage of 160 mm. Double-syringe power injector (OptiVantage DH, Mallinckrodt, USA) was used for contrast-enhanced CT scanning. The Iohexol contrast agent (370 mgI/ml, OMNIPAQUE, GE) was intravenously bolus injected into the cubital vein with the dose of 0.8 ml/kg and the injection speed of 5 ml/s. A total of 30 ml of normal saline was injected with the same speed. Dynamic volume CT scan was started 8 s after the injection, with 2-s intervals for 40 s. After a 6-s pause, it shifted to 4-s intervals (ended at 60 s). The patient was allowed to breathe gently throughout the examination.

### Post-processing

**Figure 1:**
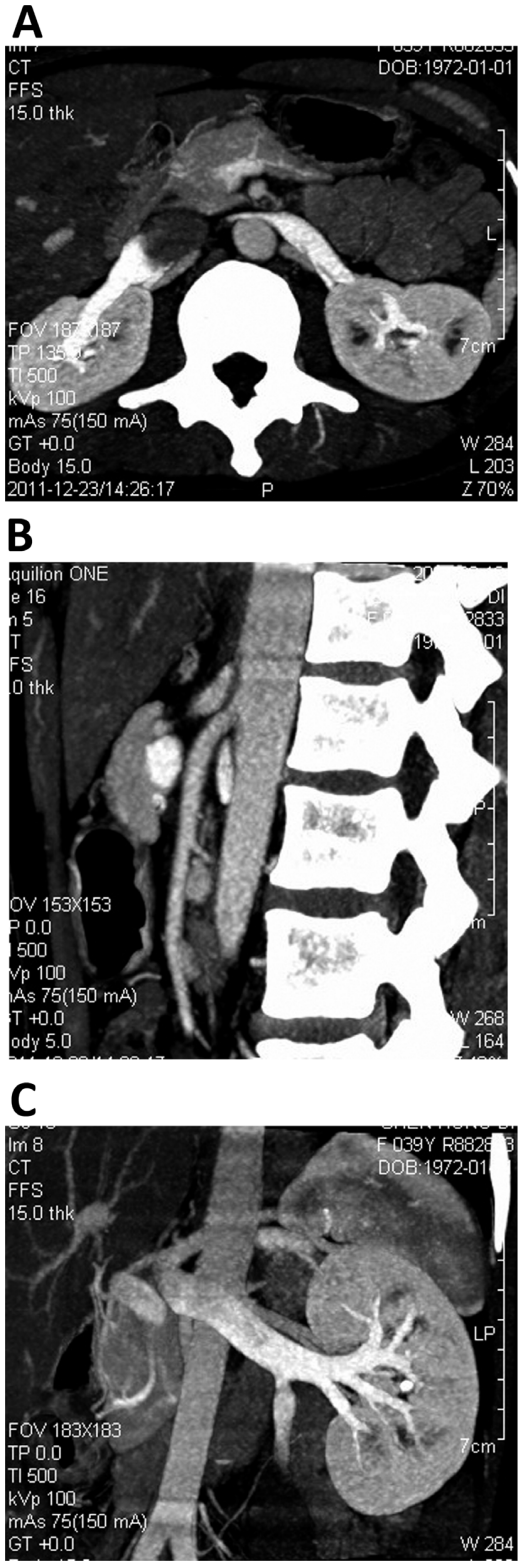
In a 38-year-old female patient with nutcracker syndrome, reconstruction included the MIP cross-sectional, sagittal and oblique coronal images.

**Figure 2:**
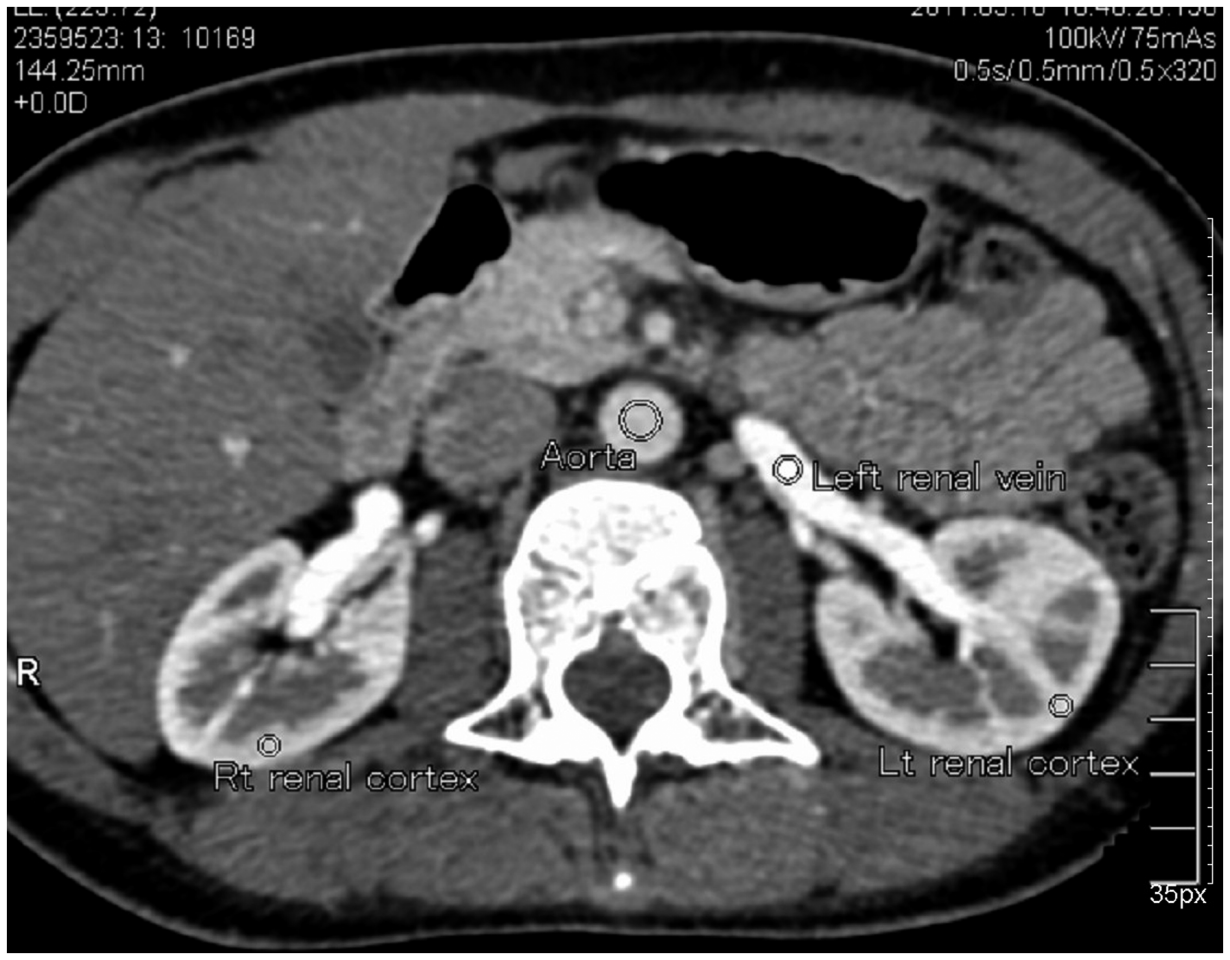
The CT perfusion regions of interest (ROI) included the aorta, left renal vein and bilateral renal cortices.

The data from dynamic volume CT scan of the 12 patients with NCS were imported for post-processing with Vitrea FX 3.0. Volume rendering, maximum intensity projection, multiple planar reformation and curved planar reformation were conducted at different phases as required. The cross-sectional areas and inner diameters at the segment of divergent or compression (where LRV passed through the SMA) were measured (Fig. 1). The original data of volume CT scanning were imported to 640-slice volume CT Aquilion One images post-processing software workstation and the Body Perfusion software of the Aquilion One was used for the automatic alignment of the original data. The abdominal aortic artery was selected to replace the renal artery as the input artery (horizontal level of left renal hilum was chosen as level of interest) and left renal vein was chosen as output vein. The regions of interest (ROI) included the aorta, renal vein and renal cortex of both kidneys (Fig. 2). The Time-density Curve (TDC) of the ROI was obtained and the corresponding renal blood flow (BF) perfusion color map was generated. The perfusion values of bilateral renal cortices were measured. The ROI was as big as possible, with pixels not less than 50 to minimize the quantum noise. Furthermore, the ROI did not include the edges of the organs to exclude the influence of partial volume effects. All the examinations were repeated three times and the mean values were calculated.

### Statistical Analysis

Statistical analysis was performed using SPSS 18.0 (SPSS Inc., Chicago, IL). The perfusion value difference between right and left renal cortexes in NCS or control groups was analyzed by paired samples *t* - test. The perfusion value difference of right or left renal cortex between the two groups was analyzed by independent sample *t* - test.

## Results

### Statistical Analysis in Age and Sex

No significant difference was found in the age (*t* = −0.503, *P = *0.618) or sex (*P = *1) between NCS and control groups.

### The Angles between the SMA and the Aorta and Left Renal Vein Measurements in NCS Patients

The angles between the SMA and the aorta were 32.3±7.6°. The inner diameters of divergent and compression parts of LRV was 10.97±1.88 mm and 3.68±1.54 mm in NCS group, respectively, with the ratio of 3.49±1.49. The cross-sectional area of these two parts was 108.08±32.11 mm^2^ and 27.63±16.38 mm^2^, respectively, with the ratio of 5.77±3.85 (Table 1).

**Table 1:** Left renal vein measurements in NCS patients (n = 12).

Location of measurement	Range	Mean	SD
The angles between the SMA and AA (°)	16.5–43.1	32.3	7.6
The smallest anteroposterior diameter of the left renal vein passing through the SMA angle (mm)	1.3–5.9	3.68	1.54
The smallest cross-sectional area of the left renal vein passing through the SMA angle (mm^2^)	4.9–49.3	27.63	16.38
The anteroposterior diameter of the widest segment of the left renal vein that does not passthrough the angle (mm)	8.9–15.5	10.97	1.88
The cross-sectional area of the widest segment of the left renal vein that does not passthrough the angle (mm^2^)	62.9–155.3	108.08	32.11
The ratio of the anteroposterior diameter at the widest and narrowest segments of theleft renal vein	1.83–6.85	3.49	1.49
The ratio of the cross-sectional area at the widest and narrowest segments of the left renal vein	1.92–13.67	5.77	3.85

Note: SD, Standard Deviation; SMA, superior mesenteric artery; AA, abdominal aorta.

### The Colored Renal Perfusion CT Images of Bilateral Renal Cortexes of the Two Groups

**Figure 3:**
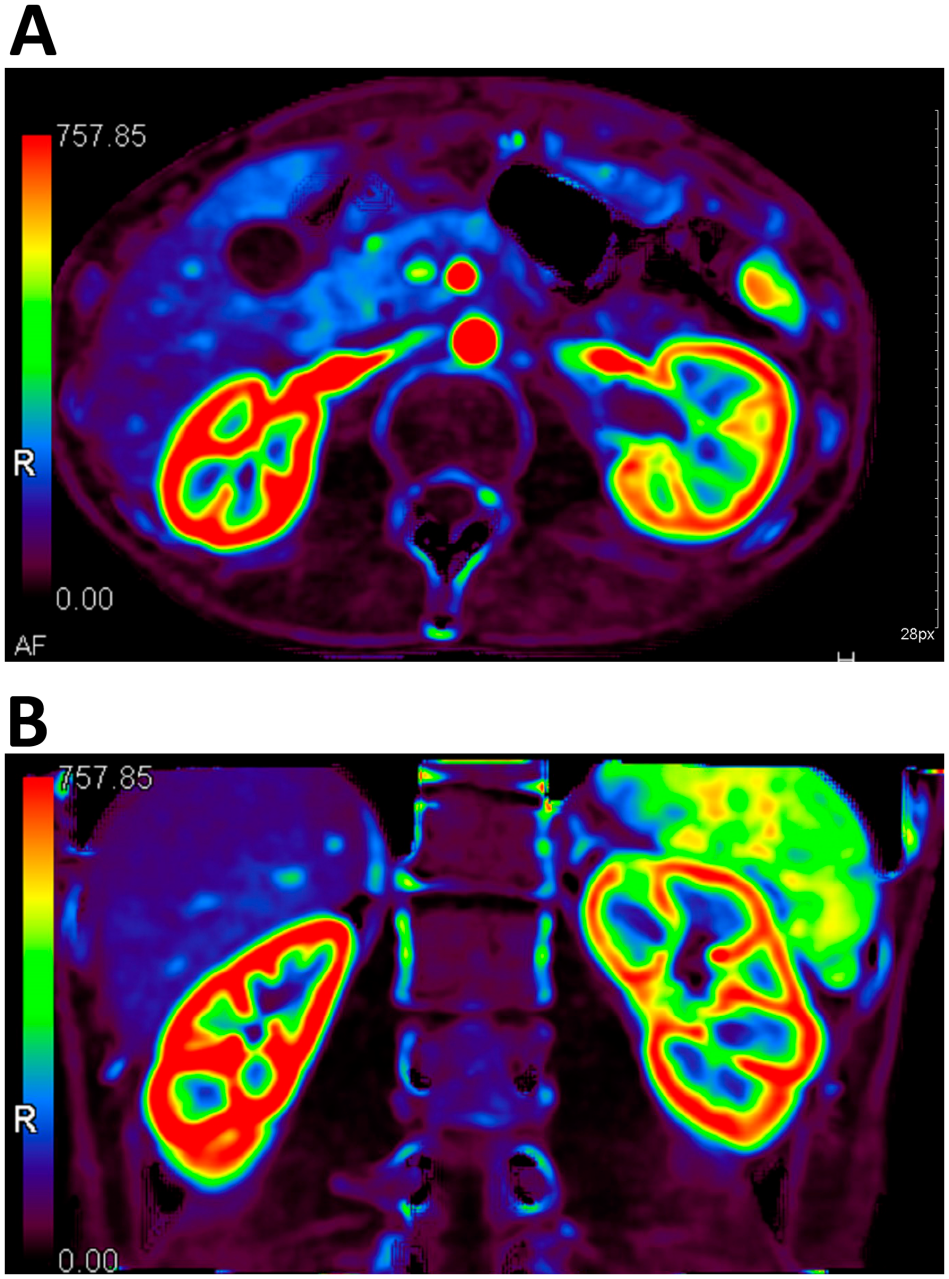
In a 32-year-old male patient with NCS, the right renal cortex was shown as a continuous intact high-perfusion red ring. The left renal cortex was less smooth and continuous and local yellow low-perfusion areas were seen.

The perfusion regions of interest (ROI) were displayed as red (the highest perfusion), yellow, green, blue and purple (the lowest perfusion) on the image processed by Body Perfusion software. The perfusion of the bilateral kidneys was uniform in control group and the renal cortexes (shown as continuous intact high-perfusion red rings). However, for patients in NCS group, the left renal cortex was less smooth and continuous and local yellow low-perfusion areas were observed. The right renal cortex was shown as a continuous intact high-perfusion red ring (Fig. 3).

### Comparison of Perfusion Values of Left and Right Cortexes in NCS and Control Groups

The perfusion values of left and right renal cortexes in control group were 323.8 and 322.9 ml·min^−1^·100 ml^−1^, respectively. For NCS group, the perfusion values of left and right renal cortexes were 350.8 and 391.1 ml·min^−1^·100 ml^−1^, respectively. The LRV stenosis was divided into severe, moderate and mild stenosis according to the ratio of the smallest area of the LRV that passed through the angle to the widest area of the LRV that did not pass through the angle. From among the 12 patients with NCS, seven had left renal vein stricture of more than 75% (severe stenosis), and the mean perfusion value of their left renal cortex was lower than that of their right renal cortex (difference = 55.1 ml·min^−1^·100 ml^−1^); four patients had left renal vein stricture of 50–75% (moderate stenosis), and the mean perfusion value of their left renal cortex was lower than that in their right renal cortex (difference = 20.8 ml·min^−1^·100 ml^−1^); one patient had left renal vein stricture of less than 50% (mild stenosis), and the left renal cortex perfusion value was 14.7 ml·min^−1^·100 ml^−1^ lower than that of the right renal cortex perfusion value (Table 2). While comparing the perfusion values of left and right renal cortexes, significant difference was found among NCS group (*t* = −4.204, *P = *0.001) but not in the control group (*t = *1.388, *P = *0.193). Similarly, while comparing the perfusion values of right or left renal cortexes between NCS and control groups, significant difference was found in the right cortex (*t = *2.752, *P = *0.012) but not in left cortex (*t = *1.099, *P = *0.284) (Table 3).

**Table 2:** Degree of stenosis of the left renal vein and renal cortical perfusion values in 12 patients.

Patient No.	Patient’s agein years	Degree ofstenosis (%)	Left renal cortical perfusion values(ml·min^−1^·100 ml^−1^)	Difference between the left and right corticalperfusion values (ml·min^−1^·100 ml^−1^)
1	38	48	311.3	−14.7
2	43	59	388.3	−5.5
3	20	61	370.2	−14.6
4	17	63	395.9	−54.2
5	63	68	365.3	−8.9
6	18	76	378.7	−25.6
7	19	79	288.1	−111.8
8	14	81	356.3	−22
9	32	89	366.7	−91.5
10	28	90	315.2	−50
11	23	90	343.9	−37.7
12	19	93	329.9	−47.3

**Table 3:** Renal cortical perfusion values in the NCS group and the control group (ml⋅min^−1^ 100 ml^−1^).

	NCS group	Control group	*t* - value	*P* - value
**Left renal** **cortex**	350.8 (288.1–395.9)	323.8 (212.3–483.5)	1.099	0.284
**Right renal** **cortex**	391.1 (326.0–458.2)	322.9 (214.2–482.6)	2.752	0.012
***t*** ** - value**	−4.204	1.388		
***P*** ** - value**	0.001	0.193		

### Doses of Radiation

For NCS group who underwent one-stop scanning with 640-slice volume CT, the mean CTDL vol was 41.4±8.4 mGy and the mean DLP was 673.3±143.1 mGycm.

## Discussion

Left renal vein (LRV) angiography has been acknowledged as the “golden standard” for NCS diagnosis. When LRV is 0.490 kPa (5 cmH_2_O) or even more higher than IVC, then LRV compression is likely to be present [1], [3]. However, this invasive method was very complex and was not well accepted by patients who did not undergo stent implantation. Color Doppler ultrasound was the most commonly used method for the clinical diagnosis of NCS. This modality could measure the anteroposterior diameter of LRV in different positions and peak flow velocity to reflect the LRV compression. As the compressed LRV was always linear or oval in shape, it is difficult to determine from the changes in anteroposterior diameters of LRV, the exact compression level [1], [3]. CT angiography (CTA) (with superior image processing software and high spatial resolution images) can clearly display the anatomy of LRV, SMA and AA as well as branch vessels. After 3-D image post-processing, the spatial structure and 3-D direction of SMA and AA can be clearly displayed. In addition, accurate measurements for anteroposterior diameter and cross-sectional areas in any area of interest can be accurately determined from CTA. On ultrasound, the compressed LRV has a linear or oval configuration; however, direct measurement of anteroposterior diameter of LRV may not well reflect the exact stenosis level. The ratio of the cross-sectional areas at the widest and narrowest segments of the LRV may provide more accurate and direct measurement of vessel luminal changes induced by LRV compression [5].

In this study, one-stop scanning of kidneys with 640-slice volume CT was performed and data were collected simultaneously within a very short time period. The spatial structures and 3-D directions of the LRV, SMA and AA were observed from different angles and aspects. In addition to measurements, we could also observe (as a result of LRV compression) the development of peri-renal and peri-ureteral venous collaterals in 5 patients and gonadal varices in 4 patients.

The CTA examination is non-invasive and has many advantages in diagnosing NCS. However, further measurement of hemodynamic changes in left kidney induced by LRV compression is required to evaluate the perfusion level and kidney function. Such information is not only critical for NCS diagnosis but also for prognosis and patient management. CT perfusion can provide a non-invasive method for evaluating abnormal blood flow in kidneys. Conventional multi-slice spiral CT provided only anatomical or functional information of the target organ in one scanning session. More CT scanning was required to provide more data, which also exposed the patients to higher doses of radiation and contrast agents. The 640-slice volume CT covered 16 cm in the z-direction and almost the whole upper abdomen was scanned by means of serial rotational acquisitions at a single location in the z-direction. Scanning with this CT provided isotropic and isophasic anatomical and functional information of all the organs in the upper abdomen or certain organs [10], [11], [12]. The long axis of each kidney was about 12–13 cm, thus one single scanning with 640-slice volume CT scanner covered the bilateral kidneys and provided the complete data for the organs. This was of great advantage, because it not only eliminated the errors caused by patients’ horizontal movements and time differences in the process of the images, but also avoided additional radiation exposures that were present in spiral scanning. Furthermore, it also overcame some of the limitations of the conventional CT perfusion imaging. Conventional CT perfusion imaging could only be performed on a certain slice and could not exclude the disturbance of respiratory artifacts [13], [14]. It can therefore be seen that 640-slice volume CT scanning is a promising method for the evaluation of hemodynamic changes in renal microcirculation.

Most of the renal blood flow goes to the cortex where the glomeruli are located. Only 6–8% of renal blood flow goes to the outer medulla and about 1–2% of the blood flows to the inner medulla and papilla. Thus, renal cortex perfusion can be used to reflect the blood flow of kidneys [15]. Paul et al. [9] reported that changes in renal cortical perfusion and TDC curve were found in patients with unilateral renal artery stenosis. In the control group, the cortical peak height and time were similar and symmetrical; and the perfusion was similar in bilateral kidneys. Our control group showed similar findings. In our study, decreased left renal cortex perfusion was observed compared with the perfusion of the right renal cortex in all 12 patients with NCS. Seven had LRV stricture of more than 75% and the mean perfusion value of their left renal cortexes was lower than that of their right renal cortexes (difference of 55.1 ml·min^−1^·100 ml^−1^); four patients had LRV stricture of 50–75% and the mean perfusion value of their left renal cortexes was lower than that of their right renal cortexes (difference of 20.8 ml·min^−1^·100 ml^−1^); one patient had LRV stricture of less than 50% (mild stenosis) and the left renal cortex perfusion value was 14.7 ml·min^−1^·100 ml^−1^ lower than that of the right renal cortex.

Evidence of abnormally low perfusion in a stenotic kidney may have predictive value for renal atrophy and may constitute an indication for revascularization [9]. The findings in our study show the degree of LRV compression correlates directly the decrease in the left renal perfusion. As shown in colored blood flow perfusion images, the perfusions of the bilateral kidneys were uniform in control group and the renal cortex was shown as continuous intact high-perfusion red rings. However, for patients in the NCS group, the left renal cortex was less smooth and continuous, and local yellow low-perfusion areas were observed, while the right renal cortex was shown as a continuous intact high-perfusion red ring. Statistical analysis also demonstrated that there was no significant difference in perfusion value of the left renal cortex between the two groups. However, a significant difference was found while comparing the perfusion value of the right renal cortex between the two groups. In our study, the perfusion values of left and right renal cortexes in control group were 323.8 and 322.9 ml·min^−1^·100 ml^−1^, respectively. For NCS group, the perfusion values of left and right renal cortexes were 350.8 and 391.1 ml·min^−1^·100 ml^−1^, respectively. The perfusion values in NCS group were higher in most cases than that in the control group. But, in NCS patients, the perfusion value significantly decreased in left renal cortex compared to that of the right renal cortex, so while comparing the perfusion values of right or left renal cortexes between NCS and control groups, significant difference was found in the right cortex but not in left cortex. The results of our study suggested that the renal cortex perfusion differed from each other in different individuals and that a patient with unilateral renal ischemia could not be diagnosed just based on the perfusion value of the renal cortex in different individuals. We suggested that the renal blood flow of the other side should be also considered as a better method for unilateral renal ischemia diagnosis.

There are two important limitations in our study. Firstly, although there appears to be a correlation between LRV stenosis and left renal perfusion, we are unable to suggest a diagnostic criterion for NCS. This is due to our study design and small sample size. Secondly, even though the radiation dosage given to our patients is lower than the reported dosage in 320-slice CT perfusion studies [10], [11], [12], the radiation dosage for one-stop scanning with 640-slice volume CT remains a great concern, especially for children.

In summary, one-stop whole-organ examination of kidneys using 640-slice volume CT can offer an opportunity to obtain the renal angiography and volume images of both kidneys, as well as the whole kidney organ perfusion imaging with a single scan. Furthermore, it reflects the tissue hemodynamic changes through the quantitative measurement of the tissue perfusion; it is easy to reveal profound hemodynamic changes of the left renal perfusion in many patients with NCS. This method can associate the physiological functions of organs with their morphological changes in order to make the examinations as easy as a “one-stop” examination. This method may provide a theoretical basis for quantitative diagnosis and clinical intervention of NCS, as well as providing a foundation for prognosis estimation of the NCS.
